# Electrophysiologic findings in patients with COVID-19 and quadriparesia in the northwest of Iran, A case series study and literature review

**DOI:** 10.22088/cjim.12.0.451

**Published:** 2021

**Authors:** Fariba Eslamian, Negar Taleschian-Tabrizi, Behzad Izadseresht, Seyyed Kazem Shakouri, Shakiba Gholian, Mohammad Rahbar

**Affiliations:** 1 Physical Medicine and Rehabilitation Research Center, Imam Reza Hospital, Tabriz University of Medical Sciences, Tabriz, Iran

**Keywords:** Acute motor axonal neuropathy, COVID-19, Corona virus, Critical illness myopathy, Critical illness neuropathy, GBS, Quadriparesis

## Abstract

**Background:**

As a global health pandemic, the novel severe acute respiratory syndrome-coronavirus 2 (SARS- CoV2) outbreak began in December 2019 which rapidly spread to more than 200 countries. Respiratory complications and fever are the most obvious symptoms. Sometimes the neurological features are superimposed on the main disease and complicate patient's status.

**Case Presentation:**

We describe 6 patients with COVID-19 and concomitant quadriparesia who underwent electrodiagnosis using EMG/NCS and results indicated 3 axonal variants of Guillain–Barré syndrome (GBS), including; 2 cases AMAN (acute motor axonal neuropathy), 1 case AMSAN (acute motor and sensory axonal neuropathy), three myopathies, including one combination of CIN/CIM (critical illness neuropathy/critical illness myopathy), one CIM and one acute polymyositis in these cases.

**Conclusion:**

Early diagnosis of the neuromuscular disorders of coronavirus could help for correct planning in the treatment of COVID-19 patients. Since GBS and inflammatory myopathies have an autoimmune basis, the immunotherapies such as IVIG, steroids, plasma exchange and other novel treatments as hemoperfusion can promise better and faster recovery in respiratory function and neuromuscular activity among COVID-19 patients who have musculature paralysis concomitantly. However, all these treatments are challenging and further clinical trials should be done to confirm the efficacy and safety of mentioned therapies.

The novel coronavirus, causing severe acute respiratory syndrome- coronavirus 2 (SARS- CoV2), emerged in Wuhan City, China, on the late December 2019 which drew significant attention and effort as a global health concern (1). World Health Organization (WHO) named it coronavirus disease-2019 (COVID-19) and declared it as pandemic on March 11, 2020 (2). On October 10^th^ 2020, approximately 45 million cases of COVID-19 were reported, including 1,189,499 death (3). In addition to respiratory tract infection symptoms, neuromuscular, and nervous system involvement are more recently identified as presentations of this disease (4). Patients with more severe infections have common co-morbidities, and tend to present more atypical symptoms, including neurological and skeletal muscle injury manifestations (5). 

Previous studies on MERS and SARS suggested a direct invasion of virus or molecular changes due to immune-mediated response causing neuromuscular disorders such as Guillain-Barre Syndrome (GBS) which could be the same in SARS-CoV2 (6-8). It is known that SARS-CoV-2 can stimulate immune reaction resulting in cytokines storming, especially Interleukin-6 (IL-6), which activates inflammatory cascade causing tissue damage (9). Central or peripheral nervous system involvement may be caused by the direct invasion of viruses or stimulating the body's innate and adaptive immune responses to infection. However, the available evidence is not in favor of high neurovirulence of SARS-CoV-2 or related coronaviruses, unlike herpes simplex virus and some enteroviruses, causing the destruction of neurons (10). On the other hand, prolonged hospitalization and mechanical ventilation can lead to secondary neuromuscular problems such as critical illness polyneuropathy (CIP) or critical illness myopathy (CIM) (11) which their occurrence was not well studied in severe cases of COVID-19 patients. This case series aims to describe the clinical and electrodiagnostic findings of six patients who had coronavirus disease and suffered from four limb muscular weakness.

## Cases presentations

**Case1**: A 52-year-old man presented with cough and dyspnea, and due to O_2_ saturation below 90%, he was admitted to ICU in a teaching hospital, a specialized center for COVID patients. Respiratory distress progression led to intubation. PCR test, as well as lung CT-scan, was positive for coronavirus. Routine anti-COVID treatment is in-line with protocols for respiratory care demanding ICU-patients, including broad-spectrum antibiotics, anticoagulant therapy with enoxaparin, vitamin D oral supplement, none steroids anti-inflammatory drugs, and corticosteroids were administered. Due to four limbs, weakness patient was referred for electrodiagnosis 18 days after admission. EMG/NCS test showed an acute motor axonal neuropathy (AMAN), classified as GBS axonal variant neuropathy. Thus, IVIG 15-20 g/day for 5 days was added to his medications plan, and after 3 weeks, the patient was extubated. After a total duration of 50 days, he was discharged with partial dyspnea and relatively good recovery, and he was advised to continue quarantine at home. Table 1 shows demographic, clinical, and para-clinical findings and table 2 shows electrodiagnostic parameters and final EDX diagnosis in this patient.

**Table 1:** Demographic and clinical characteristics of COVID -19 patients associated with quadriparesia who underwent electrodiagnsotic tests

**Patient ID**	**Age**	**sex**	**First symptoms**	**Concomitant problem (PMH)**	**Interval between disease onset-weakness onset and disease onset-EDX exam (days)**	**Level of conciseness** **(GCS score) at EDX time**	**COVID PCR test chest CT, WBC** **CPK**	**Electrodiagnostic diagnosis**	**Drug Treatment**
**#1**	52	male	coughing and respiratory distress	--	10-18	14	PCR: positive Chest CT: positive WBC:4500 lymph:18% CPK:172 CSF Protein:54mg/dl	AMAN*	IVIG+ prednisolone+ routine anti COVID
**#2**	62	male	Renal colic and fever	Nephrolithiasis, Pyelonephritis, nephrectomy and diabetes	5-15	12	PCR: positive Chest CT: positive WBC:17000 lymph:6.7%	AMAN*	IVIG+ Broad-spectrum antibiotics+ anti COVID
**#3**	76	male	Diarrhea and cough	--	5-7	15	PCR: positive Chest CT: positive WBC:14000 CRP:+3 CPK:328 CSFProtein:76mg/dl	**AMSAN†**	IVIG+ routine anti COVID
**#4**	58	male	Dry cough and respiratory dyspenea	-	20-22	10	Initial PCR: positive After 6 weeks :Neg CPK:1283 WBC:11300 Lymph:7.1%	**CIN/CIM** ^€^	IVIG+ prednisolone+ routine anti COVID
**#5**	32	female	Fever and respiratory distress	--	45-69	14	Initial PCR: positive After 8 weeks :Neg WBC:11600 CPK:450	**CIM** ^€^	routine anti COVID+ IVIG +prednisolone
**#6**	34	male	Muscular weakness and respiratory distress	Opium addict	0-30	13	Initial PCR: posetive Chest CT: positive CPK:1819 WBC:14000	**Polymyositis**	Prednisolone-hydroxychlroqiune+ routine anti COVID+ IVIG

*AMAN: acute motor axonal neuropathy,** †AMSAN:** acute motor and sensory axonal neuropathy,^ €^**CIN/CIM: **critical illness neuropathy/critical illness myopathy.

**Table 2:** Electrodiagnostic parameters among COVID-19 patients with quadriparesia

**Patient ID**	**Tibial CMAP** ^*^ **amp** ^‡ ^ **(mv)**	**Tibial CMAP** **distal latency(ms)**	**Tibial CMAP** **NCV** ^€^ **(m/s)**	**Tibial** **F-wave latency** **(ms)**	**Peroneal CMAP** ^* ^ **amp** ^‡^ **(mv)**	**Median CMAP amp** ^‡^ **(mv)**	**Median** **CMAP** ^*^ **NCV** ^€^ **(m/s)**	**Ulnar CMAP** ^*^ ** amp** ^‡^ **(mv)**	**Sural SNAP** ^† ^ **amp** ^‡^ **(µv)**	**Ulnar SNAP† amp** ^‡^ **(µv)**	**Median SNAP† amp** ^‡^ **(µv)**	**Presence** **of muscle** **fibrillation and MUAP** ^₤^ ** pattern**	**EDX** **Impression**
#1	3.8^Ω^	6.1	38.5	60.3	0.0	0.3	48	0.8	6	15	14	+2,neurogenic	AMAN
#2	3.6	5.5	42	0.0	0.0	0.7	50	1.4	3	28	17	+3, neurogenic	AMAN
#3	2.0	7.2	44	0.0	0.8	7.9	36	5.2	0.0	10	11	0, neurogenic	AMSAN
#4	1.6	6.0	49	58.5	0.0	0.8	58	1.2	0.0	17	14	+1,neuromyogenic	CIN/CIM
#5	3.4	7.4	53	48	1.8	2.8	51	2.2	18	30	28	+1,myogenic	CIM
#6	0.4	6.8	54	0.0	0.2	2.7	56	2.0	24	27	46	+3, myogenic	Polymyositis

*CMAP: compound muscle action potential, ^‡^** amp: **amplitude**,** **†SNAP: **sensory nerve action potential, ^€^**NCV**^: ^nerve conduction velocity. **Ω:** the numbers, which are below or above the reference values, are shown as bold numbers. ^₤^**MUAPs**: motor units action potentials. Average of right and left side motor or sensory nerves are presented.

**Table 3:** Diagnostic criteria for electrophysiological and clinical features of neuromuscular disorders in this study (12, 30-31)

	**Clinical picture**	**Motor NCS**	**Sensory NCS**	**Needle EMG findings**
**Classic GBS**	Acute onset of weakness and numbness in distal of lower limbs then upper limbs in adults and children. Progressive weakness ensues over the course of 2-4 weeks. Elevated CSF protein without pleocytosis is the characteristic finding. Immunologic attack against myelin sheath exists.	Prolonged distal motor latencies and slow conduction velocities associated with conduction block, absent or prolonged F-waves latencies .(within demyelination range; NCVs^€^<70%of lower limit of normal and latencies greater than 150% of upper limit of normal).	Prolonged distal sensory latencies, low amplitude SNAPs	Reduced recruitment of MUAPs^₤ ^associated with some fibrillation potentials may be seen when CMAPs are low amplitude and these signs are present between weeks 2-4 (peaking 6-15 weeks).
AMAN*	Abrupt onset of generalized weakness in adults or children. Distal muscles more affected than proximals. Cranial nerves deficit and respiratory failure requiring mechanical ventilation is seen in one-third of patients. Antecedent and positive serology of C.Jejuni is seen in 67-90%. Albuminocytologic dissociation is the rule.	Low amplitude or absent CMAPs^₤^. If obtainable, distal latencies and conduction velocities are normal or mildly reduced (axonal loss pattern)	Normal SNAPs^µ^	Increased fibrillation potentials and decreased recruitment of large, long and polyphasic MUAPs mainly in distal muscles (neurogenic pattern)
**AMSAN** ^†^	Rapidly progressive and severe generalized weakness over only a few days as opposed to a couple weeks in most AIDP patients. Facial and respiratory muscles involvement can be expected. DTRs are diminished or absent.	Low amplitude or absent CMAPs. If obtainable, distal latencies and conduction velocities are normal or mildly reduced (axonal loss pattern)	Low amplitude or absent SNAPs	Increased fibrillation potentials and decreased recruitment of large, long and polyphasic MUAPs (neurogenic pattern)
**CIN** ^‡^	Severe generalized muscle weakness, may be first recognized by inability to wean patient from ventilator. Respiratory muscle involvement is prominent. DTRs are diminished or absent. Sepsis and multiorgan failure are the primary causes. That is common among patients with staying longer than 20 days in ICU.	Low amplitude or absent CMAPs	Low amplitude or absent SNAPs	**In severe & acute form**: inability to actively recruit MUAPs with presence of profuse fibrillation potentials. **In early reinnervation**: small and polyphasic MUAPs are present and then with **recovery **large MUAPs are recruited.
**CIM** ^Ω^	Severe generalized muscle weakness, may be first recognized by inability to wean patient from ventilator. DTRs are diminished or absent CPK usually moderately elevated (up to 10 times of normal value). Other names: Acute quadriplegic myopathy, acute illness myopathy myopthy with loss of myosin or thick filaments	Low amplitude CMAPs	Normal or mildly reduced SNAPs	**In severe & acute form**: inability to actively recruit MUAPs with presence of fibrillation potentials, **In moderate involvement or recovery period:** small, short and polyphasic MUAPs, are frequently present.(myogenic pattern)

* AMAN: acute motor axonal neuropathy,^ †^** AMSAN:** acute motor and sensory axonal neuropathy, ^‡^**CIN:** critical illness neuropathy** ,**^ Ω^**CIM: **critical illness myopathy, ^€^**NCV:** nerve conduction velocity,^ ₤^**CMAP: **compound muscle action potential**,** , ^µ^**SNAP: **sensory nerve action potential, are present ^₤^**MUAPs**: motor units action potentials

**Case 2**: A 62-year-old man with a history of diabetes and nephrolithiasis was admitted to the hospital due to fever and renal colic. Due to severe pyelonephritis and several unsuccessful dialyses, he underwent nephrectomy surgery. During the hospitalization period, respiratory problems and decreased consciousness were added to the clinical picture, and CT-scan showed the signs of pulmonary involvement with COVID-19, and the patient transferred to ICU. Anti-COVID therapy, including azithromycin, hydroxychloroquine, and other medications due to the above-mentioned protocol were prescribed to him. The patient was referred for electrodiagnosis 15 days after admission due to four limbs muscular weakness. Electrodiagnostic findings were suggestive of acute motor axonal neuropathy (AMAN) or GBS axonal variant neuropathy. IVIG 20 g SD was added to the treatment plan, and broad-spectrum antibiotics due to kidney surgery, diabetes and long-term patient hospitalization were continued. After 3 months, the consciousness level of patient improved, he was still sedated and unable to tolerate *weaning *from mechanical *ventilation*; hence, he was tracheostomized and then discharged to home due to his consent. Two weeks later, his clinical status deteriorated due to uremia and decreased in O2 saturation, and he was hospitalized again, and finally, after 2 weeks passed away.

**Case 3: **A 76-year-old male patient presented to the hospital with diarrhea and coughing and a history of one-week common cold. Chest CT showed minor pulmonary changes in favor of involvement with coronavirus, and the PCR test was positive. After 5 days of admission, he could not move his legs and could not stand or walk. Therefore, electrodiagnostic tests to evaluate lower limb weakness were performed. During the EDX examination, the patient was absolutely conscious under nasal auxiliary oxygen without intubation or respiratory distress. EMG/NCS showed the mixed type axonal and demyelinating sensorimotor peripheral neuropathy, which could be related to early stages of GBS or one of its axonal variants because only 5 days passed and the findings of electrodiagnostic criteria for typical GBS was not completely fulfilled yet (12). IVIG 20 g per day for 5 days concomitant anti- COVID treatment was started for the patient, and after 2 weeks of admission, he was discharged to home with good recovery.

**Case 4**: A 46-year-old man with dry cough and dyspnea presented to emergency care department. His O_2 _saturation at admission was 78%, and the PCR test of coronavirus was positive, and lymphopenia and anemia were also detected in laboratory tests. Muscular weakness in four limbs and persistent respiratory distress caused the patient to be referred to the electrodiagnosis center in the rehabilitation department after 22 days of admission for further evaluation to determine the reason for quadriplegia. 

EMG/NCS exam showed a critical illness neuropathy concomitant with myopathy in proximal muscles (CIN/CIM), associated to serum CPK rising. Prednisolone 25 mg daily and IVIG 5 gr SD and then 3 sessions of hemoperfusion were added to baseline anti-COVID therapy, and after 35 days of admission, the patient was discharged to home with good health status.

**Case 5**: A 32-year-old female patient who was one of the laboratory sciences staff initially presented with symptoms of common cold and then suffered from fever and respiratory disturbances. At the time of admission, the PCR test was positive, and she was hospitalized in ICU; her clinical picture deteriorated with empyema and pneumomediastinum. Anti COVID-19 treatment and broad-spectrum antibiotics and then hemoperfusion was prescribed. After 70 days of admission due to systemic illness and quadriplegia, she underwent electrodiagnostic tests, and CIM was diagnosed. Long-term admission leads to disuse and muscle mass atrophy, and finally, CIM superimposed on her underlying pulmonary illness. A tracheostomy was performed for her due to prolonged admission and inability to wean from mechanical ventilation. 

At last, after 3.5 months, she was tracheostomized, repeated PCR tests were negative, infections were improved and the patient had a relatively stable condition discharged to home. Now at follow-up evaluation, she has recovered well, her tolerance without supplemental oxygen increased, and she is on the waiting list for decannulation and tracheostomy removal. 

**Case 6**: An addict 34-year-old male patient before admission suffered from four limb muscular weakness and myalgia for several days. He was admitted to a local hospital due to myopathy and pneumonia with serum CPK level raised up to 1820 IU/lit. Afterward, he was referred to a specialized center and admitted to the rheumatology department. Bilateral pulmonary consolidations in the bases of two lungs were observed regarding coronavirus involvement. 

O_2_ saturation was 92%, and serum CPK was still elevated, and the patient was transferred to ICU. 30 days after disease onset, electrodiagnostic tests were performed. The results showed an acute and ongoing myogenic process in all upper and lower limbs muscles which was suggestive of active polymyositis. Prednisolone 60 mg daily, hydroxychloroquine 200mg, IVIG 25 gr for 5 days were prescribed to him associated to other antibiotics and drugs. Two weeks later, PCR testing was negative, but the patient was still intubated and hospitalized in the ICU because of neuromuscular weakness and the inability to weave mechanical ventilation. Then, multiple complications, including pneumothorax, pulmonary thromboembolism (PTE), and consequent tracheal arterial bleeding due to full anticoagulant dose, deteriorated patient’s condition. 

At last, he passed away after a total duration of 3 months of hospitalization. All the first 5 patients had areflexia and patient 6 had hyporeflexia. CSF analysis in 2 patients with GBS variants was performed and results are shown in table 1. The summary of clinical and EDX findings of these 6 patients is indicated in tables 1-2.

This study was performed according to the ethical standards, and the local ethics committee approved it with an assigned number of IR.TBZMED.REC1399.534.

## Discussion

To categorize our findings, we encountered two types of neuromuscular dysfunction among COVID-19 patients: first; co-incidence of axonal variants of GBS associated to infection within the acute phase (less than 4 weeks of disease onset) and second; critical illness neuropathy and myopathy, which are the consequences of long term admission in the hospital which could occur in any critical and chronic systemic illness after a longstanding stay in ICU (more than 4-8 weeks of hospitalization), especially in patients with respiratory failure who need mechanical ventilation. GBS is an acute immune-mediated polyradiculoneuropathy in which the mechanism of autoimmune disorder plays an important role to create it (13). GBS is classified due to the type of nerve fibers involvement and mode of fiber injury to acute inflammatory demyelinating polyneuropathy (AIDP), acute motor axonal neuropathy (AMAN), acute motor and sensory axonal neuropathy (AMSAN) or a subtype consistent to ophthalmoplegia, ataxia, and areflexia without any weakness which is defined as Miller Fisher Syndrome (MFS) (14). 

About two-thirds of cases are associated to an antecedent infection such as Campylobacter Jejuni which is more frequent in axonal variant, and it is also reported in viral diseases such as SARS-CoV, MERC-CoV, or Zika epidemics (13-15). There were some reports of covid-19 patients who developed GBS as AMAN type following this disease (16). The electrophysiological feature of NCS in AMAN is low amplitude or unobtainable CMAPs with normal SNAPs. When CMAPs are obtained, distal latencies and conduction velocities are normal or mildly reduced. Increased fibrillation potentials and decreased recruitment of neurogenic MUAPs (motor units action potentials) could be appreciated in the needle exam (12). All of these findings were observed in electrodiagnostic tests of our sampled patients (cases 1-2).

To our knowledge, four cases of GBS due to COVID-19 were reported in Iran. Sedaghat and Karimi reported a 65-year-old man with acute progressive symmetric ascending quadriparesis who had confirmed COVID-19 diagnosis two weeks before disease onset. Electrodiagnostic studies were compatible to AMSAN or an axonal variant of GBS (16). Ebrahimzadeh et al. reported two COVID-19 cases with(acute inflammatory demyelinating polyneuropathy or classic GBS) (AIDP) (17), and Farzi et al. revealed a demyelinating type polyneuropathy in a 41- year- old male patient who developed ascending paralysis following infection with SARS-CoV-2 virus. Electrodiagnostic evaluation in this patient indicated AIDP and was treated with IVIG, which resulted in a favorable response (18). 

A systematic review about neurological manifestations of COVID-19 was published, and 82 cases with neurological disorders were identified in which 17 patients had GBS (4). Gupta A et al. suggested that whereas AIDP and demyelinating neuropathy are more common with GBS related to dengue and Zika virus, the most COVID-19-related GBS patients had Acute motor axonal neuropathy (AMAN) and AMSAN (19). This result is exactly confirmed by the current study. In this regard, from electrodiagnosis point of view, our patients showed axonal variants of GBS, including AMAN and AMSAN rather than classic GBS. Moreover, motor nerves involvement was greater than sensory nerves, and low amplitudes CMAPs were more frequent than low amplitudes SNAPs. In contrast, two recent systemic reviews showed the results against above conclusion, and they indicated patterns more compatible with a demyelinating polyradiculoneuropathy versus axonal type polyneuropathies (4, 20). Anyway, COVID-19 is a novel, challenging infective disease, and its complications need to be explored more in the future. Accordingly, all GBS subtypes (AIDP, AMAN, AMSAN, MFS) can be expected with COVID-19; therefore, screening of ganglioside antibodies to assess autoimmunity may be helpful (21). 

In this case series, we diagnosed three myopathies among COVID-19 patients, including a combination of CIM/CIN, pure CIM, and acute polymyositis. The last patient (case 6) was admitted with a clinical picture of polymyositis initially and after hospitalization infection of coronavirus and pneumonia superimposed on the underlying myositis. The former patients (cases 4-5) were admitted to ICU because of COVID-19, and they were involved with CIM/CIN or pure CIM several weeks later.

Skeletal muscle injury is defined as a patient having myalgia and elevated serum creatine kinase level above 200 IU/L. The last studies indicated that patients with COVID-19 and muscle injury had significantly higher levels of creatine kinase (median, 400.0 IU/L), regardless of their severity (5). Mao et al. concluded that this injury may be due to the direct effect of the virus on muscle tissue. The other possible mechanism proposed is the infection-mediated immune response which cause elevated pro-inflammatory cytokines in serum, resulting in skeletal muscle damage; however, the association of SARS-CoV-2 binding with angiotensin- converting enzyme-2 (ACE-2) receptors in muscle is other probable mechanism, but there is still need for more investigation. Patients with a muscle injury had lower lymphocyte counts, higher neutrophil counts, higher C-reactive protein and D-dimer levels, and increased blood coagulation function as well (5).

Zhang et al. reported a 38-year-old man with COVID-19 and myalgia in which lab findings showed extremely increased CPK (>42,670 U/L), as well as elevated inflammatory markers indicating a myositis associated to rhabdomyolysis. It was discovered that this complication was due to SARS-CoV-2 (22). Viral myositis can be caused by viruses such as influenza A and B, human immunodeficiency virus (HIV), hepatitic B and C, and herpes simplex virus (23). Beydon et al. reported a case of myositis secondary to covid-19 in a patient. Symptoms appeared suddenly on walking with diffuse myalgias, increased CPK level, and proximal lower limb muscle weakness, causing him to fall. Fever occurred at day 4, and chest involvement of COVID-19 emerged on day 10 after the initial presentation. MRI showed bilateral edema in the external obturator muscle and quadriceps, suggesting myositis (24). The history of this patient was in line with our case 6, who was predominantly presented with polymyositis manifestation and followed by pulmonary involvement, which could emphasize the importance of non-specified musculoskeletal presentation in early diagnosis of COVID-19. Critical illness polyneuropathy (CIP) involving sensorimotor nerves and critical illness myopathy (CIM) involving skeletal muscles are frequent complications which can occur in 25–45% of critically ill patients, especially those with long term ICU hospitalization, mechanically ventilated ones, serious non-neurological medical reasons including acute or chronic renal failure, COPD exacerbation, pulmonary thromboembolism (PTE), cerebral and gastrointestinal hemorrhage, complications of ischemic heart diseases, or multiple organ failure (11, 25). 

The underlying pathology may be microcirculatory dysfunction leading to hypoperfusion or hyper-inflammatory status which can damage motor neurons integrity (26). COVID-19 leads to severe ARDS (acute respiratory distress syndrome), and the necessity for mechanical ventilation was reported from 26% to 47.3 % in severe case (27, 28) which indicates the possibility of CIP and CIM occurrence is considerable, CIP/CIM are not well studied in COVID-19 yet. CIN and CIM tend to appear later than GBS in our study, and it was mentioned that the difference among them is important (29). Clinical features and electrodiagnostic findings of AMAN, AMASAN, CIM, and CIN are presented in table 3 (12, 30-31). There is no established treatment for CIN and CIM, the treatment of the underlying disease is vital. IVIG was recently noticed as an adjuvant treatment of COVID-19, and early studies showed the promising effects of IVIG at the therapeutic dose of 0.3–0.5 g/kg to improve the general condition of COVID-19 patients, especially in early administration in critically ill patients (31- 33). Moreover, combining IVIG with a moderate-dose of corticosteroids may improve patients outcomes (34). Relatively favorable outcomes were achieved from using IVIG in our study; however, the efficacy is not thoroughly confirmed yet, and there is the potential of the exaggerated inflammatory response (33).

 Other novel treatment options opposing immune dysregulation in COVID-19, such as extracorporeal hemoperfusion, are a feasible option to purify blood to stop cytokine storm (35). Plasma exchange could also be beneficial by removing cytokines and preventing pathologic coagulation, and maintaining microcirculation (36). Plasma from patients recovered from COVID-19 or convalescent plasma containing antibodies against SARS-CoV2 has shown promising results in patients with severe COVID-19 (37). Thus, electrodiagnostic tests and early diagnosis of acute neuropathies or critical illness neuromyopathies could emphasize using novel treatments even more, which could be beneficial for both conditions. 

In conclusion to conclude, we reported six cases with COVID-19 associated to quadriparesia who underwent electrodiagnostic tests, and it was found that two cases had AMAN, one AMASN, one CIN/CIM, one pure CIM, and one polymyositis. Immunotherapies such as steroids, plasma exchange, IVIG, and novel procedures could be good and promising treatments in GBS variants, which may be beneficial for pulmonary lesions of COVID-19 at the same time. Therefore, early diagnosis of acute neuropathies or myopathies could help better planning of treatment among patients with COVID-19 who have neuromuscular weakness concomitantly. Nonetheless, all these treatments are challenging and further clinical trials are required to confirm the efficacy and safety of mentioned therapies.
